# An Experimental and Theoretical Study of Dye Properties of Thiophenyl Derivatives of 2-Hydroxy-1,4-naphthoquinone (Lawsone)

**DOI:** 10.3390/ma14195587

**Published:** 2021-09-26

**Authors:** Matías Monroy-Cárdenas, Oscar Forero-Doria, Ramiro Araya-Maturana, Maximiliano Martínez-Cifuentes

**Affiliations:** 1 Instituto de Química de Recursos Naturales, Universidad de Talca, Talca 3460000, Chile; matias.monroy@utalca.cl; 2 Departamento de Ciencias Básicas, Facultad de Ciencias, Universidad Santo Tomás, Talca 3460000, Chile; oforero@santotomas.cl; 3 Departamento de Química Orgánica, Facultad de Ciencias Químicas, Universidad de Concepción, Edmundo Larenas 129, Concepción 4070371, Chile

**Keywords:** lawsone derivatives, dye-sensitized solar cells, metal-free organic dyes, photovoltaic parameters

## Abstract

A prospective study of the dye properties of non-toxic lawsone thiophenyl derivatives, obtained using a green synthetic methodology allowed for the description of their bathochromic shifts in comparison to those of lawsone, a well-known natural pigment used as a colorant that recently also has aroused interest in dye-sensitized solar cells (DSSCs). These compounds exhibited colors close to red, with absorption bands in visible and UV wavelength range. The colorimetric study showed that these compounds exhibited a darker color than that of lawsone within a range of colors depending on the substituent in the phenyl ring. Computational calculations employing Density Functional Theory (DFT) and Time-Dependent Density Functional Theory (TD-DFT), showed that the derivatives have lower excitation energies than lawsone, while the alignment of their frontier orbitals regarding the conduction bands of TiO_2_ and ZnO and the redox potential of the electrolyte I^−^/I_3_^−^ suggests that they could be employed as sensitizers. The study of the interactions of the lawsone and a derivative with a TiO_2_ surface model by different anchoring modes, showed that the adsorption is thermodynamically favored. Natural bond orbital (NBO) analysis indicates a two-center bonding (BD) O-Ti as the main interaction of the dyes with TiO_2_.

## 1. Introduction

Since the components of the quinone/hydroquinone redox couple are easily inter-convertible and closely balanced energetically [1,2], they serve as important links in electron transport processes during metabolic pathways and many biological reactions [3], also explaining their use in industrial applications [4,5,6,7]. Among naturally occurring quinones, lawsone (2-hydroxy-1,4-naphthoquinone), a red-orange pigment, present in henna, has long been used for the coloration of the skin, hair, and textile materials [8]. Important reasons for the use of lawsone as a dye are the ease of obtaining it and the absence of environmental problems related to its use. It has been demonstrated to not be mutagenic in catalase mutant Escherichia coli, both in the absence and presence of metabolic activation [9]. On the other hand, it has been suggested that its low cytotoxicity is probably mediated, at least in part, by the release of reactive oxygen species [9]. However, extensive research in the field of synthetic dyestuffs has led to a drastic decline in the use of natural dyes because of their limited range of colors among other disadvantages. Despite the advantages that synthetic compounds offer in this area, several synthetic colorants are ultimately environmentally deleterious. On the other hand, besides its use as a textile colorant, lawsone has aroused a recent interest in the fields of protein crystallography [10] and dye-sensitized solar cells (DSSCs). In DSSCs, it has been employed as a dye sensitizer (which acts as both a light absorber and electron donor) [11], where it is attached to a semiconductor anode surface, which consists of an inorganic mesoporous oxide layer acting as electron acceptor. When sunlight is absorbed by the dye, an ultrafast electron transfer from the dye’s first excited state to the conduction band states of the semiconductor occurs. Then, the oxidized dye is neutralized by the reduced form of a redox mediator, which can be either a liquid electrolyte redox couple (usually iodide/triiodide or metal complexes) or a solid-state positive charge (hole) transporter. Finally, the oxidized redox mediator is neutralized at the cathode to close the circuit. A major requirement of an efficient solar cell sensitizer is to have an extended UV/Vis absorption spectrum closely matching that of solar radiation [12]. In this way, the dye sensitizer plays an essential role in achieving a high-power conversion efficiency of the DSSC [12]. The development of dye sensitizer can be grouped in two main sets: (a) dyes based on the ruthenium (II) complex and (b) metal-free organic donor–acceptor dyes [13]. The first group contains the metal ruthenium, which has a high environmental impact and is expensive. The second group of dyes presents major advantages due to their inexpensive cost and tunable molecular design [13]. It is important to note that among the second group, natural pigments as sensitizers have emerged as an interesting option [14,15,16,17,18,19,20]. One of the main ways by which higher power conversion efficiency in DSSCs can be achieved is the development of a sensitizer capable of using photons from the near-infrared spectral region [21]. Some studies on the performance of lawsone in DSSCs indicated the energy conversion efficiency to be 0.93% with an absorption spectrum range of 200–410 nm. Moreover, co-sensitization with lawsone has been used to improve the performance of betanin solar cells [22,23]. Although the above shows that lawsone represents a promising prospect, it is important to identify derivatives that have a wider absorption range at wavelengths closer to the near-infrared spectral region.

Lawsone and its conjugated base are involved in a pH-dependent equilibrium. The acidic solution of lawsone is colorless, although, with increased pH values, an absorption maximum of around 450 nm develops, and this gives rise to the characteristic orange color. The solution in acetonitrile is very pale yellow and presents an absorption band at 333 nm, but it turns intense yellow upon dilution with water. However, the absorptions of the UV/Vis spectrum of lawsone in dimethyl sulfoxide are observed at the 296, 339, 416, and 448 nm wavelengths [24].

There are few examples in the literature regarding the tuneability of the spectroscopy properties of lawsone using structural modifications. One example is the tuning of the original color of lawsone using cocrystallization with pyridine derivatives [25].

Thus, this study aims to assess the impact of structural modifications on dye characteristics. Lawsone thiophenyl derivatives are easily accessible via a green synthetic methodology and have shown low toxicity in a previous study [26]. An experimental–theoretical approach was used to carry out a detailed description of the properties of these derivatives as dyes (Table 1), and the potential application in colorimetry and solar cells compared with that of lawsone is discussed. 

## 2. Results

### 2.1. Colorimetric Study 

**Figure 1:**
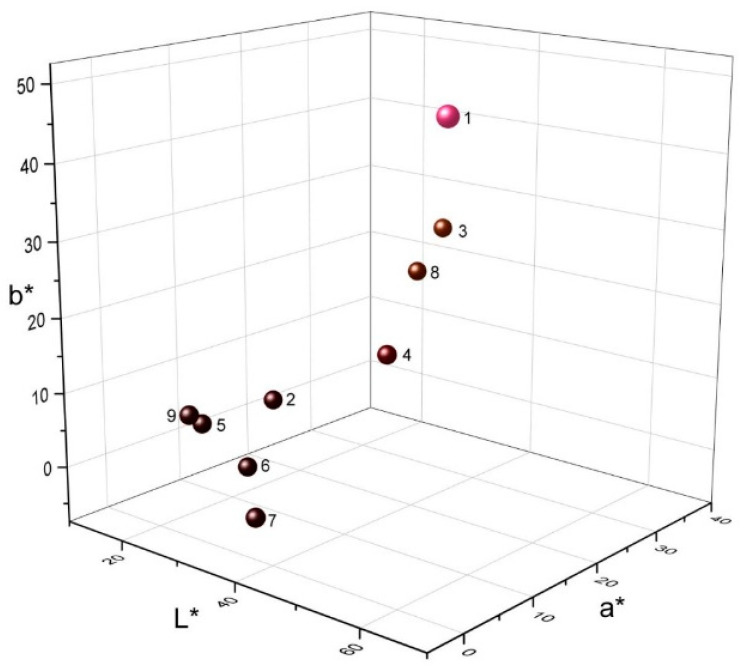
Three-dimensional chromaticity diagram of the different thio-lawsone derivatives.

The CIE Lab system was used to evaluate the color parameters and the color differences of the thiophenyl derivatives of lawsone; the results are shown in Table 2. According to a* and b* values, their colors were present in the yellow-red quadrant. These compounds showed L* values < 40, indicating that they have darker colors than those of lawsone (1) (L* > 60). The color differences (ΔE) were significant at 51.8–71.2 (Figure 1) due to the incorporation of the differently substituted thiophenyl moieties in lawsone structure **1**. The bromo derivatives, such as **5** and **6**, showed the highest ΔE with values of 71.2 and 70.4, respectively, while fluoro derivatives **7** and **9** showed the lowest values of 65.6 and 67.4, respectively. The L* value of **1** was compared with the published value of the henna dye solution [27].

Lawsone analogs, such as the natural anthraquinones emodin and dermocybin, exhibit differences in color; i.e., emodin dyed polyamide is brownish-orange, while dyed with dermocybin is wine red, but on polyester, the colors are totally different: bright yellow and bright reddish-orange, respectively [28]. Similar results were obtained for different styryl and azo-lawsone dyes of synthetic origin, exhibiting brown (CIELab: 25.057, 13.577, and 17.516) and orange (CIELab: 63.554, 36.699, and 67.159) colors on polyester, respectively, while on nylon, both exhibited darker colors (L* < 40) in the yellow-red quadrant, which agree with our results [29,30]. Moreover, polymers based on lawsone have been obtained with color differences. For example, Mahkam et al. (2014) synthesized a series of polymers derived from lawsone with acryloyl chloride, methacrylic acid, acrylic acid, and acrylonitrile to obtain different polymers using free-radical copolymerization. The resulting polymers presented colors in the yellow-red quadrant [31] in a similar manner to the thiophenyl lawsone derivatives reported here.

Generally, the color of dyes depends on the nature of their coupled components [32]; consequently, this series of lawsone derivatives shows that it is possible to obtain a wide gamma of colors by replacing the substituent in the phenyl ring of this kind of lawsone thiophenyl derivative, thereby suggesting potential industrial uses. 

### 2.2. UV/Vis Spectroscopy

**Figure 2:**
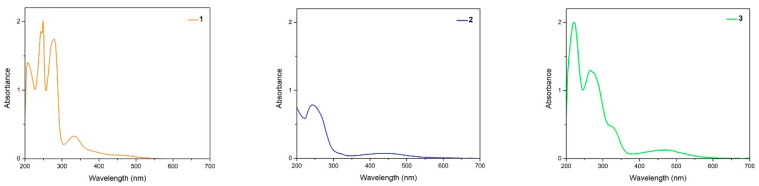
UV/Vis absorption spectra of compounds **1**, **2**, and **3** in methanol. Spectra for all compounds are presented in supporting information.

Figure 2 and Table 3 resume experimental UV/Vis results. Figure 2 shows the absorption bands from 200 to 700 nm for lawsone and its thiophenyl derivatives 2 and 3 (spectra for all compounds are presented in supporting information). Lawsone dyes exhibited peaks at range 208–334 nm. The band at 334 nm is attributed to C=O and OH groups [33], and its long tail in the visible region is responsible for the yellow-orange color of lawsone [34].

UV/Vis spectra of lawsone derivatives showed bands at range 205–478 nm. The latter indicates a bathochromic shift from 334 nm (lawsone) to values over 450 nm. This displacement to the visible region corresponds with a color close to red for all derivatives.

### 2.3. Theoretical Calculations

#### 2.3.1. Electronic and Spectroscopic Study

To gain insight into the molecular structure and the effect of modifications on the electron distribution and energy levels of lawsone derivatives, we carried out DFT and TD-DFT calculations. To evaluate different functionals and their suitability to this kind of calculation, we chose three DFT functionals, B3LYP, M06-2X, and HSE2PBE, in addition to the 6-311+G(d,p) basis set to perform the calculations. To evaluate the accuracy of the employed functionals, we compared the HOMO energy of lawsone obtained experimentally [35] with values obtained using these functionals. The calculated EHOMO was −7.50 eV for HSE2PBE, −8.80 eV for M06-2x, and −7.36 eV for B3LYP. B3LYP gave a value closer to the experimentally reported value (−5.21 eV) than the other functionals did. Therefore, we continue our study with the results obtained when using this function. 

**Figure 3:**
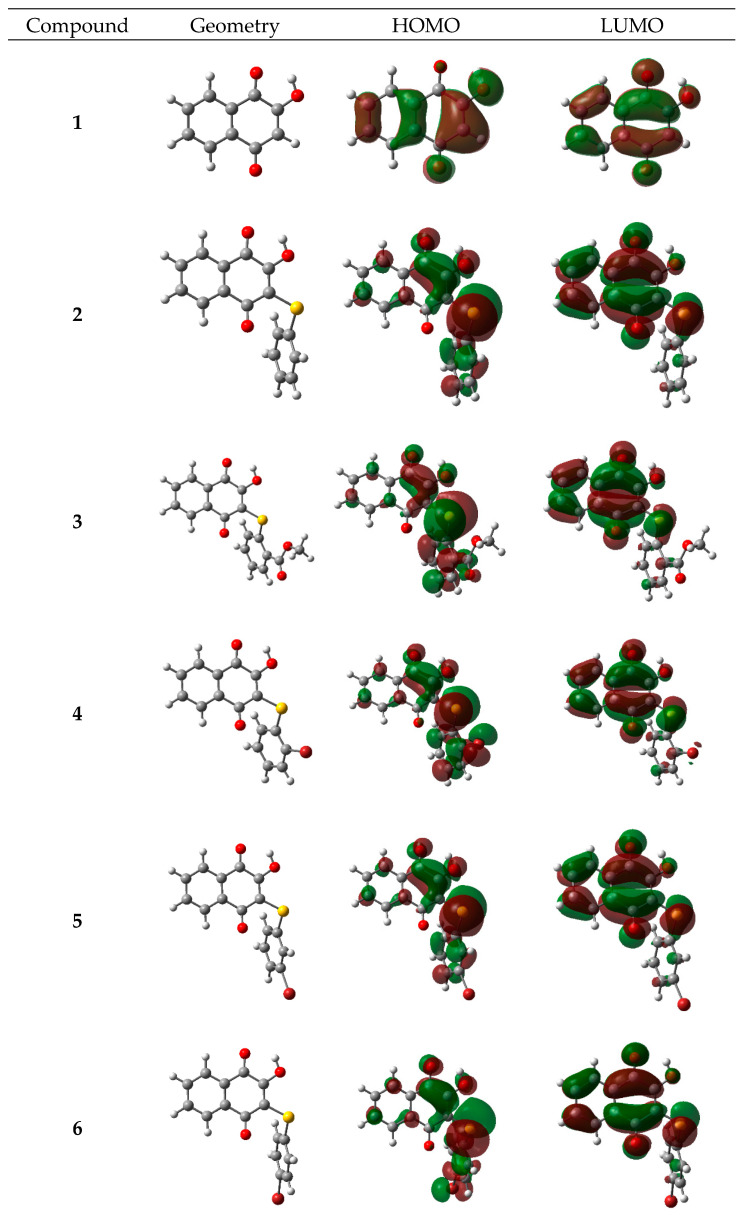

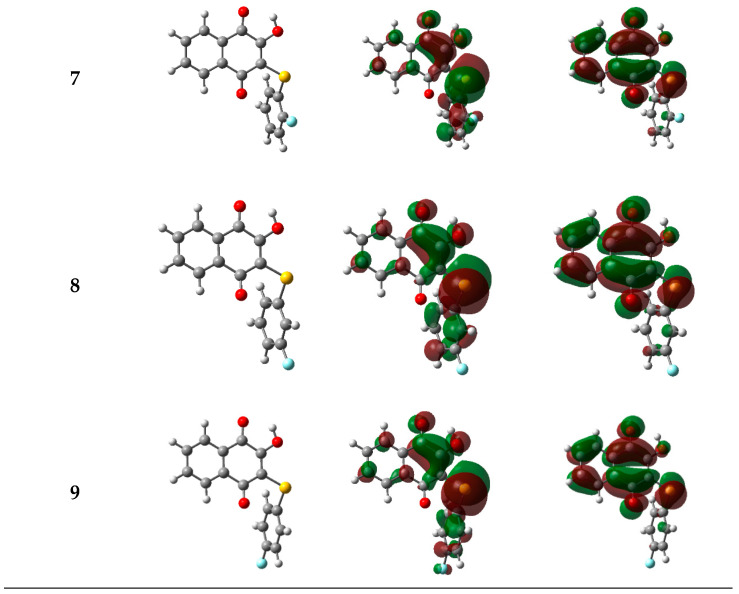
Optimized geometries and electronic densities corresponding to the HOMO and LUMO energy levels for all studied molecules.

The optimized geometries, as well as HOMO and LUMO distribution for lawsone and its thio derivatives, are presented in Figure 3.

All derivatives present a CSC angle at about 103°, and dihedral CCSC at about 50°, which leads to the formation of the aromatic ring of thiophenyl moiety, and the quinonic ring does not become coplanar. This structural feature can lead to a decline in the charge transfer between both sides of the molecule and also generates an anti-aggregation effect [36]. It has been widely studied that aggregation of dyes that can interact by π-π interactions tend to negatively affect their performance in DSSCs [37]. Aggregation of dyes molecules in a face-to-face alignment, as occur in p-p interactions, lead to hypsochromic absorption shift (H-aggregate) [37]. The latter avoids that dye can absorb in the longer-wavelength region of the visible and near-infra-red (NIR) spectrum, which are a desirable part of the spectrum. It has been experimentally tested that for organic dyes, aggregation, when they are on TiO_2_ surface leads to quenching of the excited states of the dyes, resulting in low efficiency of electron injection [38,39]. Besides, aggregation favor charge recombination between the injected electrons in the semiconductor and the oxidized redox mediator in the electrolyte resulting in a reduced Voc value [39,40,41].

In the case of lawsone, its coplanarity favors their potential aggregation by π-π interactions. Therefore, non-planar derivatives which prevent this kind of aggregation can lead to an improvement in their dye sensitizer performance [42]. 

Electronic distribution of frontier orbitals demonstrates that for all derivatives, the HOMO is localized mainly on the thiophenyl moiety and partially spread on the quinone ring. On the other hand, also for all derivatives, LUMO is fully localized on the quinone ring and sulfur atom with almost no presence on the aromatic ring of the thiophenyl moiety. These results show that overlap between HOMO and LUMO occurs in the lower extension for these derivatives when compared with that for lawsone.

To understand the experimental UV/Vis results, TD-DFT calculations were carried out for all compounds. Table 4 presents the excitation energies (Eex), wavelength (λ), oscillator strengths (f), light-harvesting efficiency (LHE), and the corresponding electronic transition for selected relevant excited state (full data is in Table S1 of SI). As has been previously described for lawsone, the most probable transitions, in both vacuum and solution (methanol), fall in the UV region, and they occur from the ground state to excited states 4 and 5, which can be assigned mostly to the H-2→L and H-3→L MOs [14]. On the other hand, for all these derivatives, the most probable transitions, in both vacuum and solution (methanol), fall in both the visible and UV regions; occur from the ground state to excited states 1 and 8 (7 for Cpd 3), respectively; and can be assigned mostly to the H→L and H→L+1 MOs. The LHEs calculated for lawsone in vacuum are similar to those previously described [14], and they are considered rather low, as in the general case of natural dyes [43,44]. In methanol, we noted that LHE increased more than in dimethyl sulfoxide and dichloromethane according to s previous report [14]. LHEs for thiophenyl derivatives of lawsone decrease slightly, but they demonstrate absorption in the visible range, which benefits their potential performance in DSSCs. Comparisons among theoretical and experimental UV peaks are shown in Table S2 of the Supplementary Material.

To study the alignment of energy levels, which define the overall conversion efficiency of DSSCs, we considered the alignment of the frontier Mos energy levels of the compounds with valence and a conduction band edge of the semiconductors TiO_2_ and ZnO. Table 5 lists the MOs, excitation energies E_ex_, energy gaps E_gap_, and excited-state oxidation potentials (ESOPs). ESOPs have been proposed as appropriate for the representation of excited states, and they were calculated from occupied energy levels and relevant excitation energies [14,45,46]. E_ex_ is the most important factor in the evaluation of the efficiency of DSSCs, as a sensitizer with a low E_ex_ facilitates light absorption in the long-wavelength range [14]. We noted that thiophenol derivatives present lower E_ex_ than lawsone, which indicates that they could be better sensitizers.

**Figure 4:**
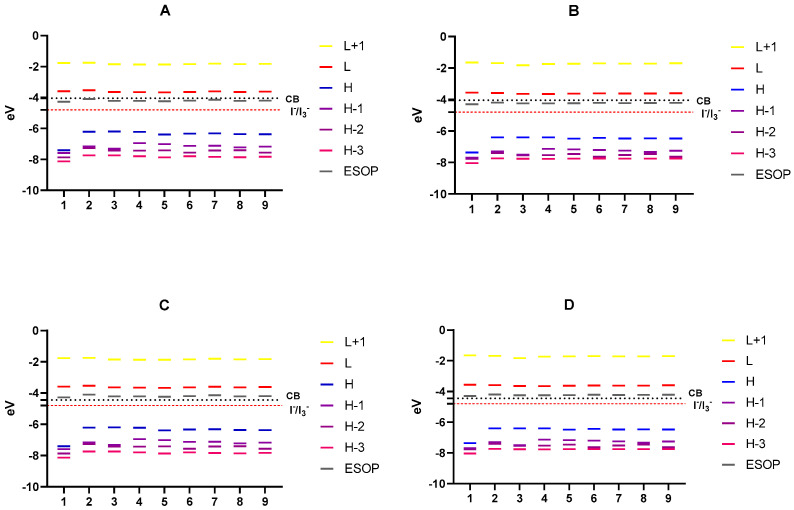
Level alignment of each compound regarding TiO_2_ ((**A**) in vacuum and (**B**) in methanol) and ZnO ((**C**) in vacuum and (**D**) in methanol). Alignment regarding electrolyte I^−^/I_3_^−^ is shown in each case. Values for TiO_2_ and ZnO have been obtained from references [48,49].

Energy diagrams of the most relevant MOs of the species in both the gas phase and solution are shown in Figure 4. E_LUMO_ of a good photosensitizer dye should lie slightly above the conduction band (CB) edge of the semiconductor to ensure electron injection from the excited dye [47], while E_HOMO_ should lie under the redox potential of the electrolyte for dye regeneration by accepting electrons from the electrolyte [23]. We used a TiO_2_ (CB = −4.05 eV [48]) ZnO (CB = −4.45 [49]) semiconductor in addition to the electrolyte I^−^/I_3_^−^ (−4.80 eV [50]) to evaluate our compounds. As displayed in Figure 4, all thiophenyl derivatives of lawsone meet the criteria of energy level alignment to be appropriate photosensitizers for TiO_2_ and ZnO semiconductors. In the case of TiO_2_, the gap between CB and E_LUMO_ is lower than that for ZnO, which suggests that these compounds perform well as sensitizers in DSSC with TiO_2_. Besides, E_HOMO_ for all derivatives lie below the redox level of the I^−^/I_3_^−^ electrolyte, allowing electron transfer to the dye from the electrolyte.

#### 2.3.2. Adsorption of Lawsone and 3-Thiophenyl-lawsone on a TiO_2_ Model

**Figure 5:**
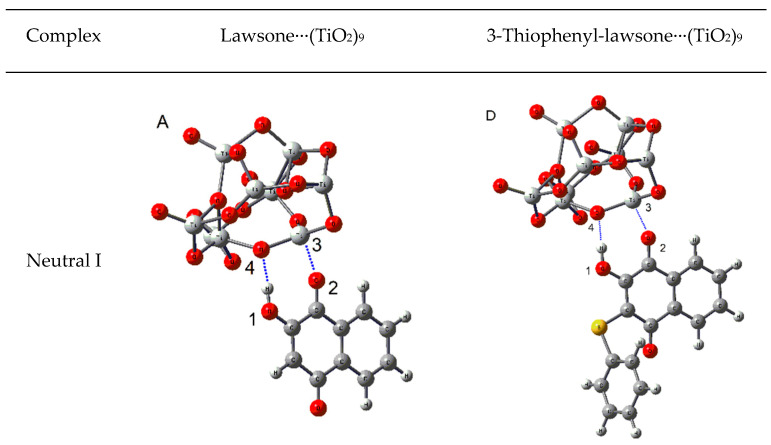

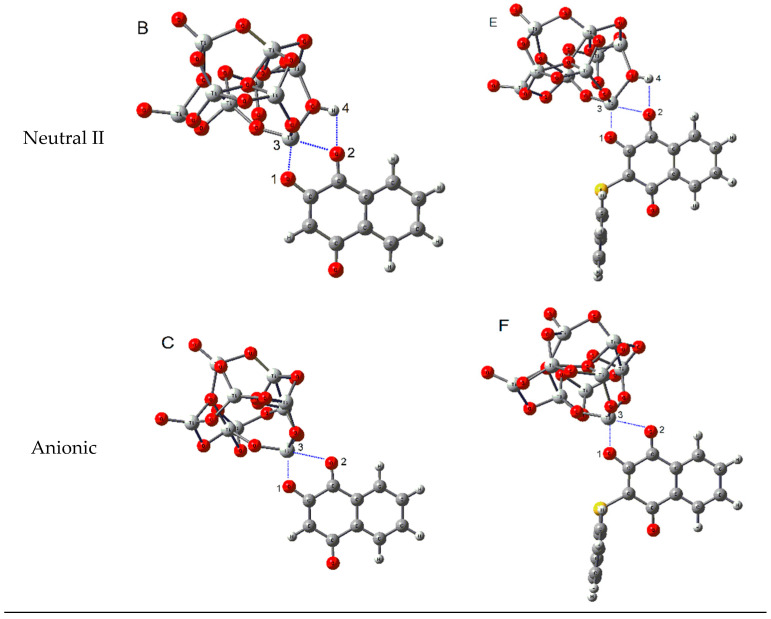
Optimized geometries for the complexes (**A**) neutral lawsone∙∙∙(TiO_2_)_9_, (**B**) neutral deprotonate lawsone∙∙∙(TiO_2_)_9_, (**C**) anionic deprotonated lawsone∙∙∙(TiO_2_)_9_, (**D**) neutral 3-thiophenyl-lawsone∙∙∙(TiO_2_)_9_, (**E**) neutral deprotonated 3-thiophenyl-lawsone∙∙∙(TiO_2_)_9_, and (**F**) anionic deprotonated 3-thiophenyl-lawsone∙∙∙(TiO_2_)_9_. Calculations at B3LYP DFT level with basis set 6-311G(d,p) for C, H, S, and H atoms, and LanL2DZ for Ti atoms.

An essential requisite for an organic dye can effectively acts as a sensitizer in DSSCs, is the anchoring to the surface of the semiconductor. The latter can be achieved by incorporating different polar functional groups in the structure of sensitizer (as carboxylic acid, amines, ketones, among others). Prinzisky et al. studied the performance of a hydroxy-anthraquinone imine as a dye for DSSCs [51], where the hydroxyl and imine groups acting anchoring the molecule to the TiO_2_ semiconductor surface. Recently, Sreeja and Pesala studied the performance of natural dyes indigo, lawsone, and betanin as co-sensitizer for solar cells [23]. They associated one carbonyl group of the lawsone as an anchor group to the surface of TiO_2_. However, lawsone possesses a hydroxyl group in the alpha position to one of the carbonyls of the quinone ring, which can additionally contribute to anchoring the molecule to the surface of the semiconductor. Considering this background, we studied the interactions between lawsone and 3-thiophenyl-lawsone with a TiO_2_ surface model by DFT calculations. The adsorption of the dyes was simulated with a (TiO_2_)_9_ cluster, based on previous works of Sánchez de Armas et al. [52,53], who showed that a group of (TiO_2_)_9_ is large enough to reproduce adequately the electronic properties of a dye-TiO_2_ system. There are different ways to adsorb a dye onto a TiO_2_ surface, depending on the conditions used to prepare DSSC [37]. Considering the above, we explored three possible anchoring modes for both molecules, depending on the protonation state of the hydroxyl (Figure 5). The first mode corresponds to a not dissociate state of hydroxyl (neutral I), the second mode corresponds to a dissociate state, where the hydroxyl proton is transferred to an oxygen of TiO_2_ surface (neutral II), and the third mode corresponds to a dissociate state where the hydroxyl proton is transferred to another species with basic character, potentially present in the medium (anionic).

For the first mode (neutral I), we found that two interactions are responsible for the anchoring: an intermolecular hydrogen bond (IHB) between the -OH in the dye and an oxygen in the TiO_2_ cluster, along with an interaction between the oxygen of a carbonyl group. The distance of O1-H1∙∙∙O3 was 1.70 Å for both complexes. In the case of O2∙∙∙Ti3 interaction, the distance was slightly shorter for the 3-thiophenyl-lawsone∙∙∙(TiO_2_)_9_ complex compared to lawsone∙∙∙(TiO_2_)_9_ (2.00 vs. 2.03 Å). For the second mode (neutral II), a bidentate interaction between both neighbor oxygen in the dye with one Ti on the surface, along with a potential IHB between oxygen protonated in the cluster and one of the oxygen in the dye, are responsible for the adsorption. The latter mode is similar to it found when carboxylic acid groups binding to TiO_2_ surface [54,55,56]. Distances for intermolecular interactions O1∙∙∙T3, O2∙∙∙T3, and O2∙∙∙H4 were shorter for the complex 3-thiophenyl-lawsone∙∙∙(TiO_2_)_9_ compared to those for complex lawsone∙∙∙(TiO_2_)_9_ (1.89 versus 1.92 Å, 2.01 versus 2.28 Å, and 2.02 versus 2.17 Å, respectively). The third mode (Figure 5C,F), with anionic deprotonated species, showed a similar configuration to the second mode, with a Ti of cluster interacting with two neighbor carbonyl in the dyes. For lawsone∙∙∙(TiO_2_)_9_ complex the O1∙∙∙Ti3 distance was shorter compared to those of 3-thiophenyl-lawsone∙∙∙(TiO_2_)_9_ complex (1.94 versus 2.47 Å, respectively). On the other hand, the O2∙∙∙Ti3 distance was shorter 3-thiophenyl-lawsone∙∙∙(TiO_2_)_9_ compared to those of lawsone∙∙∙(TiO_2_)_9_ complex (2.43 versus 2.47 Å, respectively).

To evaluate the anchoring of the studied dyes on the TiO_2_ surface quantitatively, we calculated the adsorption energies (E_ads_) for the complexes as follows [57,58]:

(1) $$E_{\mathrm{ads}}=E_{\mathrm{dye}-{\left({\mathrm{TiO}}_{2}\right)}_{9}}-E_{\mathrm{dye}}-E_{{\left({\mathrm{TiO}}_{2}\right)}_{9}}$$

where $E_{\mathrm{dye}-{\left({\mathrm{TiO}}_{2}\right)}_{9}}$ correspond to the energy of complex, $E_{\mathrm{dye}}$ to the energy of the isolated dye, and $E_{{\left({\mathrm{TiO}}_{2}\right)}_{9}}$ to the energy of isolated (TiO_2_)_9_ cluster. Table 6 presents the values of E_ads_ for each complex, as well as their HOMO, LUMO, and gap energies (E_gap_). All complexes presented negatives E_ads_, indicating that anchoring of these dyes is termodynamically favorable. Between the two neutral adsorption mode, the second (bidentate) was more favorable for both dyes. Comparing between lawsone and 3-thiophenyl-lawsone, the latter presents a more negative value, indicating a stronger interaction with the TiO_2_ cluster. Anionic complexes present the most negative E_ads_ for both dyes (C and F), indicating that they interacts stronger with TiO_2_ cluster than the neutral forms. Unlike the second mode of adsorption (the most favorable between neutrals), lawsone presented a complex more stable than 3-thiophenyl-lawsone. The value of E_ads_ obtained for complexes (−17.79 to −97.39 kcal/mol) indicates in all cases a strong enough interactions that allow one to suggest that these dyes are chemisorbed on the surface of TiO_2_ [57,59].

To analyze the nature of the interaction between anchoring atoms in the dyes with the atoms on the surface of the TiO_2_ cluster, we carried out a natural bond orbital (NBO) analysis for the most stable neutral complexes B and E, and for anionic C and F [60]. The results showed that all complexes studied present a two-center bond of type BD for O1-Ti_3_, which explains the strong E_ads_. The results also showed a less strong donor-acceptor interaction between a LP of O_2_ with Ti_3_ atoms in the cluster for C and F. Additionally, an interaction LP of O_2_ with H4 in the TiO_2_ cluster for B and E, which is characteristic of hydrogen bonds [60].

## 3. Materials and Methods

### 3.1. Synthesis 

Compounds **1**–**7** were obtained using an on-water methodology previously described [26]. ^1^H and ^13^C NMR spectra were obtained from a spectrometer operating at either 400.13 MHz (^1^H) or 100.61 MHz (^13^C) at 300 K. All melting points were uncorrected and were determined using Electrothermal 9100 apparatus (Cole-Parmer, Stone, UK). Silica gel 60 (230–400 mesh ASTM) and TLC aluminum sheets silica gel 60 F254 were used for flash-column chromatography and analytical TLC, respectively. 

### 3.2. Color Evaluation by Nix Pro Sensor

CIELab coordinates (L*, a*, b*, C*, and h°, where L* denotes lightness, a the red/green value, b* the yellow/blue value, C* the saturation value, and h° is the hue) [45] were calculated from the reflectance data of the 10° observer and daylight.

The color differences between the derivatives of lawsone and lawsone were calculated as follows:

ΔE = √(ΔL*)^2^ + (Δa*)^2^ + (Δb*)^2^ (2)

### 3.3. Measurement of the UV/Vis Spectrum

Methanol (analytical grade) from PanReac AppliChem (Castellar del Vallès, Barcelona, Spain) and lawsone from Acros Organics (Beel, Antwerp, Belgium ) were used without further purification procedures. The absorption spectra of compounds in the methanolic solution were recorded using a Double Beam UV/VIS spectrophotometer Rayleigh—UV2601 (Beijing Beifen-Ruili Analytical Instrument, Beijing, China). A solvent blank was measured before each recorded spectrum. All measurements were performed at ambient temperature in standard quartz cuvettes of a 1 cm optical path.

### 3.4. Computational Calculations

The geometries of the studied compounds were optimized at the B3LYP, M06-2X, and HSE2PBE in addition to the 6-311+g(d,p) basis set. The electronic absorption spectra of the molecules in vacuum and methanol (MeOH) were simulated using time-dependent density functional theory (TD-DFT) at the B3LYP/6-311+G(d,p) level. The solvent effect was evaluated based on the polarizable continuum model (PCM) [46]. Calculations of the dyes attached to a cluster of (TiO_2_)_9_ were carried out following a known methodology [58,61] at DFT B3LYP with basis set 6-311G(d,p) for C, H, S, and O atoms, and LanL2DZ for Ti atoms. No imaginary vibrational frequencies were found at the optimized geometries, indicating that they are the true minima of the potential energy surface.

Density Functional Theory (DFT) and Time-Dependent Density Functional Theory (TD-DFT) calculations were performed using the Gaussian 09 (Gaussian, Inc., Wallingford, CT, USA) program package [47]. Natural Bond Orbital (NBO) calculations were carried out using NBOpro 6.0 (University of Wisconsin, Madison, WI, USA) program package [62]. All simulated absorption spectra were analyzed using GaussSum 3.0 [63].

## 4. Conclusions

The results of this study showed that, by linking lawsone with substituted benzenethiols, it is possible to obtain compounds exhibiting a gamma of colors with bathochromic shifts to a color close to red when compared with that of the parent compound. The inclusion of thiophenyl moiety in lawsone allows for absorption in a wide range of visible and UV wavelength range, compared with not substituted lawsone. Computational calculations employing Density Functional Theory (DFT) and Time-Dependent Density Functional Theory (TD-DFT) showed that the compounds presented lower excitation energies than lawsone, while the alignment of their frontier orbitals regarding the conduction bands of TiO_2_ and ZnO and the redox potential of the electrolyte I^−^/I_3_^−^ suggests that they may be employed as sensitizers, like lawsone. Geometrical analysis showed that the incorporation of thiophenyl substituents on lawsone structure led to a loss of coplanarity, which favor an anti-aggregation behavior, which is a factor that improves the open-circuit photovoltage (Voc) when dyes tend to form H-aggregation, as occur commonly in organic dyes with aromatic rings. The study of the interactions of the lawsone and 3-thiophenyl-lawsone with a TiO_2_ surface model by different anchoring modes, showed that the absorption is thermodynamically favored. Natural bond orbital (NBO) analysis indicates a two-center bond (BD) O-Ti as the main interaction of the dyes with TiO_2_, both in neutral and anionic state. 

## Figures and Tables

**Table 1:** Compounds studied in this work [26].

Cpd	1	2	3	4	5	6	7	8	9
X	Lawsone	H	*o*-CO_2_Me	*o*-Br	*m*-Br	*p*-Br	*o*-F	*m*-Br	*p-Br*
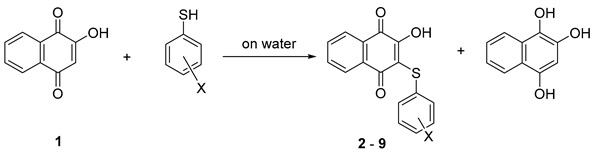									

**Table 2:** Colorimetric properties of thio derivatives of lawsone.

Compound	L*	a*	b*	c*	h°	Δ*E*
**1**	65.01	1.76	51.64	51.67	88.04	-
**2**	18.70	17.15	3.50	17.50	11.55	69.1
**3**	30.30	33.78	25.39	42.26	36.93	54.1
**4**	36.62	19.29	12.96	23.24	33.90	51.8
**5**	15.19	9.52	1.95	9.71	11.56	71.2
**6**	21.34	11.22	−3.34	11.70	343.41	70.4
**7**	31.12	4.69	−5.40	7.15	310.97	65,6
**8**	27.90	31.58	19.31	37.24	31.23	58.0
**9**	17.07	6.38	4.71	7.93	36.42	67.4

**Table 3:** Experimental UV/Vis spectrum in methanol.

Thio-Lawsone Derivatives									
	1	2	3	4	5	6	7	8	9
λ exp (nm)	208	270	222	213	212	207	208	208	205
	243	459	268	267	266	264	251	210	265
	249		273	465	467	478	280	252	277
	279		325				463	279	473
	334		474					465	

**Table 4:** Excitation energies (E_ex_), wavelength (λ), oscillator strengths (f), light-harvesting efficiency (LHE), and the corresponding electronic transition for each relevant excited state.

Vacuum						Methanol					
Excited State N°.	E_ex_, eV	λ, nm	f	LHE	Electronic Transition	Exited State No.	E_ex_, eV	λ, nm	f	LHE	Electronic Transition
Cpd 1						Cpd 1					
ES 4	3.69	336	0.058	0.125	H-2→ L (91%)	4	3.57	348	0.075	0.159	H-2→L (47%)
ES 5	4.31	288	0.177	0.334	H-3→ L (88%)	5	3.20	295	0.249	0.436	H-3→L (92%)
Cpd 2						Cpd 2					
ES 1	2.11	587	0.052	0.112	H→L (98%)	1	2.20	562	0.059	0.127	H→L (98%)
ES 8	3.93	315	0.172	0.327	H→L + 1 (88%)	8	4.13	300	0.205	0.376	H→L + 1 (96%)
Cpd 3						Cpd 3					
ES 1	1.99	624	0.053	0.114	H→L (99%)	1	2.15	577	0.059	0.127	H→L (99%)
ES 7	3.79	327	0.196	0.364	H→L + 1 (77%)	7	3.90	318	0.201	0.370	H→L + 1 (96%)

**Table 5:** Energies of molecular orbitals, excitation energies E_ex_, energy gaps E_g_, and excited-state oxidation potentials (ESOPs) for each relevant transition.

Vacuum						Methanol					
MO	E_MO_ eV	Transition	E_ex_	E_g_	ESOP	MO	E_MO_ eV	Transition	E_ex_	E_g_	ESOP
Cpd 1						Cpd 1					
H-3	−8.13	H-3→L	4.31	4.54	−3.83	H-3	−8.04	H-3→L	3.20	4.48	−4.84
H-2	−7.87	H-2→L	3.69	4.28	−4.18	H-2	−7.76	H-2→L	3.57	4.20	−4.19
H	−7.4	H→L	3.13	3.81	−4.27	H	−7.36	H→L	3.06	3.80	−4.30
L	−3.59					L	−3.56				
L + 1	−1.76					L + 1	−1.64				
Cpd 2						Cpd 2					
H-4	−7.84	H-4→L	3.67	4.31	−4.17	H-3	−7.74	H-3→L	3.55	4.15	−4.19
H-1	−7.15	H-1→L	2.77	3.62	−4.38	H-1	−7.29	H-1→L	2.88	3.70	−4.41
H	−6.21	H→L	2.11	2.68	−4.10	H	−6.40	H→L	2.20	2.81	−4.20
L	−3.53					L	−3.59				
L + 1	−1.75					L + 1	−1.69				
Cpd 3						Cpd 3					
H-6	−8.11	H-6→L	3.94	4.47	−4.17	H-3	−7.76	H-3→L	3.53	4.12	−4.23
H-5	−7.93	H-5→L	3.65	4.29	−4.28	H-1	−7.51	H-1→L	2.95	3.87	−4.56
H	−6.2	H→L	1.99	2.56	−4.21	H	−6.40	H→L	2.15	2.76	−4.25
L	−3.64					L	−3.64				
L + 1	−1.85					L + 1	−1.83				
Cpd 4						Cpd 4					
H-4	−7.92	H-4→L	3.65	4.27	−4.27	H-3	−7.77	H-3→L	3.53	4.13	−4.24
H-1	−6.95	H-1→L	2.76	3.30	−4.19	H-1	−7.14	H-1→L	2.91	3.50	−4.23
H	−6.22	H→L	2.01	2.57	−4.21	H	−6.40	H→L	2.15	2.76	−4.25
L	−3.65					L	−3.64				
L + 1	−1.86					L + 1	−1.74				
Cpd 5						Cpd 5					
H-4	−7.94	H-4→L	3.66	4.28	−4.28	H-3	−7.76	H-3→L	3.54	4.14	−4.22
H-2	−7.41	H-2→L	2.81	3.75	−4.60	H-2	−7.46	H-2→L	2.91	3.84	−4.55
H	−6.39	H→L	2.15	2.73	−4.24	H	−6.49	H→L	2.25	2.87	−4.24
L	−3.66					L	−3.62				
L + 1	−1.86					L + 1	−1.72				
Cpd 6						Cpd 6					
H-1	−7.12	H-1→L	2.75	3.48	−4.37	H-3	−7.75	H-3→L	3.53	4.14	−4.22
H	−6.33	H→L	2.13	2.69	−4.20	H-1	−7.20	H-1→L	2.85	3.59	−4.35
L	−3.64					H	−6.44	H→L	2.23	2.83	−4.21
L + 1	−1.84					L	−3.61				
						L + 1	−1.71				
Cpd 7						Cpd 7					
H-4	−7.89	H-4→L	3.65	4.29	−4.24	H-3	−7.76	H-3→L	3.54	4.14	−4.22
H-1	−7.11	H-1→L	2.75	3.51	−4.36	H-1	−7.24	H-1→L	2.88	3.62	−4.36
H	−6.31	H→L	2.17	2.71	−4.14	H	−6.48	H→L	2.25	2.86	−4.23
L	−3.6					L	−3.62				
L + 1	−1.8					L + 1	−1.71				
Cpd 8						Cpd 8					
H-4	−7.93	H-4→L	3.66	4.29	−4.27	H-3	−7.76	H-3→L	3.55	4.15	−4.21
H-2	−7.4	H-2→L	2.81	3.76	−4.59	H-2	−7.46	H-2→L	2.92	3.85	−4.54
H	−6.36	H→L	2.15	2.72	−4.21	H	−6.47	H→L	2.25	2.86	−4.22
L	−3.64					L	−3.61				
L + 1	−1.84					L + 1	−1.71				
Cpd 9						Cpd 9					
H-1	−7.17	H-1→L	2.75	3.56	−4.42	H-3	−7.74	H-3→L	3.54	4.15	−4.20
H	−6.37	H→L	2.17	2.76	−4.20	H-1	−7.26	H-1→L	2.84	3.67	−4.42
L	−3.61					H	−6.48	H→L	2.27	2.89	−4.21
L + 1	−1.82					L	−3.59				
						L + 1	−1.69				

**Table 6:** Adsorption energies (E_ads_), HOMO energies (E_HOMO_), LUMO energies (E_LUMO_) and gap HOMO-LUMO energies (E_gap_) for the six complexes A–F.

Complex	E_HOMO_ (eV)	E_LUMO_ (eV)	E_gap_ (eV)	E_ads_ (kcal/mol)
(A) Neutral lawsone∙∙∙(TiO_2_)_9_	−7.97	−4.84	3.13	−19.94
(B) Neutral deprotonated lawsone∙∙∙(TiO_2_)_9_	−7.74	−4.42	3.32	−44.60
(C) Anionic deprotanated lawsone∙∙∙(TiO_2_)_9_	−5.08	−1.61	3.47	−97.39
(D) Neutral 3-thiophenyl-lawsone∙∙∙(TiO_2_)_9_	−6.92	−4.68	2.24	−17.79
(E) Neutral deprotonated 3-thiophenyl-lawsone∙∙∙(TiO_2_)_9_	−6.62	−4.30	2.32	−46.30
(F) Anionic deprotanated 3-thiophenyl-lawsone∙∙∙(TiO_2_)_9_	−4.28	−1.69	2.59	−80.30

## Data Availability

Data Sharing Not applicable.

## Supplementary Materials

The following are available online at https://www.mdpi.com/article/10.3390/ma14195587/s1, Table S1: Excitation energies (E_ex_), wavelength (λ), oscillator strengths (f), light-harvesting efficiency (LHE), and the corresponding electronic transition for each relevant excited state, Table S2: Proposed assignation of experimental and calculated UV peak.
