# Supplementary material for: An Experimental and Theoretical Study of Dye Properties of Thiophenyl Derivatives of 2-Hydroxy-1,4-naphthoquinone (Lawsone)
Source: Materials (Basel). 2021 Sep 26;14(19):5587. doi: 10.3390/ma14195587 (PMC8509387; doi:10.3390/ma14195587)

Supplementary Information

An Experimental and Theoretical Study of Dye Properties of Thiophenyl-Derivatives of 2-Hydroxy-1,4-Naphthoquinone (Lawsone)

Matías Monroy-Cárdenas,[1] Oscar Forero-Doria,[2] Ramiro Araya-Maturana[1]*, and Maximiliano Martínez-Cifuentes[3]*

[1] Instituto de Química de Recursos Naturales, Universidad de Talca, Talca, Chile.
[2] Departamento de Ciencias Básicas, Facultad de Ciencias, Universidad Santo Tomás, Talca, Chile.
[3] Departamento de Química Orgánica, Facultad de Ciencias Químicas, Universidad de Concepción, Edmundo Larenas 129, Concepción, Chile.
* Correspondence: maxmartinez@udec.cl (M. M-Cif.), raraya@utalca.cl (R. A-M.)

1. Tables

Table S1. Excitation energies (E_{ex}), wavelength (λ), oscillator strengths (f), light-harvesting efficiency (LHE), and the corresponding electronic transition for each relevant excited state.

Vacuum						Methanol					
Excited state N°.	E_{ex}, eV	λ, nm	f	LHE	Electronic transition	Exited state No.	E_{ex}, eV	λ, nm	f	LHE	Electronic transition
**Cpd 1**						**Cpd 1**					
ES 2	3.13	396	0.010	0.023	H → L (86%)	2	3.06	405	0.007	0.016	H → L (83%)
ES 4	3.69	336	0.058	0.125	H-2→ L (91%)	4	3.57	348	0.075	0.159	H-2→L (47%)
ES 5	4.31	288	0.177	0.334	H-3→ L (88%)	5	3.20	295	0.249	0.436	H-3→ L (92%)
ES 7	5.11	243	0.178	0.337	H→L+1 (85%)	7	5.14	241	0.258	0.448	H→L+1 (78%)
ES 8	5.56	223	0.014	0.032	H-3→L+1 (59%),	8	5.60	221	0.006	0.014	H-3→L+1 (74%)

Cpd 2

ES 1	2.11	587	0.052	0.112	H→L (98%)
ES 2	2.77	447	0.009	0.021	H-1→ L (58%)
ES 6	3.67	338	0.028	0.061	H-4→L (77%)
ES 8	3.93	315	0.172	0.327	H→L+1 (88%)

Cpd 3

ES 1	1.99	624	0.053	0.114	H→L (99%)
ES 6	3.65	339	0.017	0.039	H-5→L (51%)
ES 7	3.79	327	0.196	0.364	H→L+1 (77%)
ES 9	3.94	315	0.026	0.058	H-6→L (53%)

Cpd 4

ES 1	2.01	617	0.054	0.117	H→L (99%)
ES 2	2.76	450	0.007	0.015	H-1→L (79%)
ES 6	3.65	340	0.022	0.050	H-4→L (69%)
ES 8	3.83	324	0.170	0.324	H→L+1 (83%)

Cpd 5

Cpd 2

1	2.20	562	0.059	0.127	H → L (98%)
2	2.87	432	0.009	0.021	H-1→L (64%)
6	3.54	350	0.066	0.141	H-3→L (74%)
8	4.13	300	0.205	0.376	H → L+1 (96%)

Cpd 3

1	2.15	577	0.059	0.127	H→L (99%)
6	3.53	351	0.055	0.119	H-3→L (54%)
7	3.90	318	0.201	0.370	H→L+1 (96%)
9	4.12	301	0.054	0.117	H→L+2 (93%)

Cpd 4

1	2.15	576	0.060	0.129	H → L (99%)
2	2.91	426	0.007	0.016	H-1→L (51%)
6	3.53	351	0.044	0.096	H-3→L (46%)
8	4.06	305	0.190	0.354	H → L+1 (95%)

Cpd 5

ES 1	2.15	575	0.055	0.120	H→L (98%)	1	2.25	550	0.063	0.135	H→L (98%)
ES 2	2.81	441	0.008	0.017	H-2→L (69%)	2	2.92	425	0.007	0.016	H-2→L (70%)
ES 6	3.66	338	0.041	0.089	H-4→L (84%)	6	3.54	350	0.055	0.119	H-3→L (59%)
ES 8	3.98	311	0.165	0.316	H→L+1 (90%)	8	4.17	297	0.217	0.393	H→L+1 (95%)
**Cpd 6**						**Cpd 6**					
ES 1	2.13	583	0.052	0.113	H→L (98%)	1	2.23	556	0.060	0.129	H→L (98%)
ES 2	2.76	450	0.012	0.028	H-1→L (68%)	2	2.85	435	0.010	0.023	H-1→L (72%)
ES 8	3.96	313	0.171	0.325	H→L+1 (91%)	5	3.53	352	0.045	0.098	H-3→L (55%)
						8	4.15	299	0.219	0.396	H→L +1 (95%)
**Cpd 7**						**Cpd 7**					
ES 1	2.17	571	0.048	0.105	H→L (98%)	1	2.25	552	0.056	0.121	H→L (98%)
ES 2	2.75	452	0.011	0.024	H-1→L (75%)	2	2.88	430	0.009	0.021	H-1→L (53%)
ES 6	3.65	340	0.033	0.074	H-4→L (57%)	6	3.54	351	0.060	0.129	H-3→L (68%)
ES 8	4.01	309	0.161	0.309	H→L+1 (92%)	8	4.18	297	0.215	0.390	H→L+1 (96%)
**Cpd 8**						**Cpd 8**					
ES 1	2.15	577	0.052	0.114	H→L	1	2.25	552	0.060	0.129	H→L

					(98%)						(98%)
ES 2	2.81	441	0.007	0.017	H-2→L (69%)	2	2.92	425	0.007	0.016	H-2→L (68%)
ES 6	3.66	338	0.037	0.082	H-4→L (85%)	6	3.55	350	0.052	0.113	H-3→L (58%)
ES 8	3.98	312	0.169	0.323	H→L+1 (89%)	8	4.17	298	0.217	0.393	H→L+1 (96%)
**Cpd 9**						**Cpd 9**					
ES 1	2.17	571	0.048	0.105	H→L (98%)	1	2.27	547	0.056	0.121	H→L (98%)
ES 2	2.75	452	0.011	0.024	H-1→L (75%)	2	2.84	437	0.010	0.023	H-1→L (78%)
ES 6	3.65	340	0.033	0.074	H-4→L (57%)	5	3.54	350	0.073	0.155	H-3→L (85%)
ES 8	4.01	309	0.161	0.309	H→L+1 (92%)	8	4.20	295	0.205	0.376	H→L+1 (96%)

Table S2. Proposed assignation of experimental and calculated UV peak

Compound	λ exp (nm)	λ theor (nm)	Electronic transition
1	208	221	H-3→L+1
	243	241	H → L+1
	249	295	H-3→ L
	279	348	H-2→L
	334	405	H → L
2	270	300	H → L+1
	459	562	H → L
3	222	318	H → L+1
	268	351	H-7→ L

B3LYP/6-311+G(d,p)

	273	353	H-8→ L
	325	420	H-4→ L
	474	577	H → L
4	213	305	H → L+1
	267	351	H-3→ L
	465	576	H → L
5	212	297	H → L+1
	266	350	H-3→ L
	467	550	H → L
6	207	299	H → L+1
	264	352	H-3→ L
	478	556	H → L
7	208	297	H → L+1
	251	351	H-3→ L
	280	366	H-4→ L
	463	552	H → L
8	208	298	H → L+1
	210	316	H-7→ L
	252	350	H-6→ L
	279	353	H-7→ L
	465	552	H → L
9	205	295	H → L+1
	265	350	H-3→ L
	277	350	H-3→ L
	473	547	H → L

2. Optimized geometries

B3LYP/6-311+G(d,p)

Compound 1

0 1

C	-4.49067300	-2.05812100	-0.29107800
C	-4.27247900	-0.68821600	-0.39709100
C	-3.01134900	-0.16307600	-0.10381600
C	-1.96522100	-1.00888500	0.29816000
C	-2.19430700	-2.37969100	0.40454500
C	-3.45211400	-2.90136100	0.10805900
H	-5.46747500	-2.46935900	-0.51753100
H	-5.06274800	-0.01371500	-0.70297400
H	-1.38023800	-3.01902300	0.72234700
H	-3.62435300	-3.96834600	0.19168100
C	-0.60634800	-0.46460300	0.64258400
O	0.24682800	-1.19728800	1.10720900
O	-3.60921000	2.10175000	-0.56514000
O	-1.26244400	3.11635700	-0.08422300
H	-2.14036600	3.44292800	-0.36307100
C	-2.76300900	1.28304400	-0.22320400
C	-1.38951200	1.79441100	0.05746900
C	-0.35449100	0.98214100	0.40680000
H	0.59762901	1.41246757	0.63742205

Compound 2

0 1

C	4.31301600	-1.85215500	0.34581400
C	4.00133300	-0.50284400	0.47950100
C	2.71972000	-0.05244000	0.15303600

C	1.74653400	-0.95226900	-0.30959000
C	2.06892400	-2.30175300	-0.44309900
C	3.34703900	-2.74918400	-0.11372500
H	5.30604600	-2.20556700	0.59802200
H	4.73396100	0.21255700	0.83240200
H	1.31027700	-2.98296200	-0.80764000
H	3.59189000	-3.80003700	-0.21840500
C	0.36810800	-0.48619700	-0.69182900
O	-0.41418400	-1.25740100	-1.21514400
O	3.15234700	2.23214300	0.69383700
O	0.76202000	3.10673900	0.15405700
H	1.60641300	3.48055200	0.47354800
S	-1.59607000	1.55442700	-0.83365900
C	-2.70167700	0.33673200	-0.10485000
C	-3.69180000	-0.23342600	-0.90570700
C	-2.64690500	0.04228100	1.25962000
C	-4.62518000	-1.09951600	-0.33967000
C	-3.56900800	-0.84246300	1.81332100
H	-1.89312100	0.50703700	1.88425700
C	-4.56258300	-1.41148200	1.01712700
H	-5.39228000	-1.54213600	-0.96508000
H	-3.51941900	-1.07499800	2.87129100
H	-5.28449600	-2.09272400	1.45283600
C	2.37259000	1.37113100	0.29998300
C	0.98049000	1.79935500	-0.01684200
C	0.01184600	0.93251600	-0.42286200
H	-3.71830100	-0.01411600	-1.96605400

Compound 3

0 1

C	-5.37072700	-1.21543100	0.73349900
C	-4.90747200	-0.07629200	0.08359100
C	-3.53792300	0.20180800	0.06481900
C	-2.62846100	-0.66060500	0.69819100
C	-3.10283800	-1.79905000	1.34812300
C	-4.46859600	-2.07476600	1.36394600
H	-6.43195800	-1.43460200	0.75081500
H	-5.58763800	0.60573000	-0.41163700
H	-2.39039000	-2.45204100	1.83647900
H	-4.83169400	-2.96122500	1.87141300
C	-1.15190900	-0.37371000	0.70634400
O	-0.39561800	-1.08155900	1.34440500
O	-3.74582800	2.21078500	-1.20686300
O	-1.20526500	2.74761900	-1.30786200
H	-2.03719600	3.14288900	-1.63512800
S	1.06057300	1.22320700	-0.03822600
C	1.82983900	-0.27280000	-0.67851500
C	3.11570000	-0.68695300	-0.27095500
C	1.14707800	-1.03851900	-1.63116300
C	3.64776500	-1.87538500	-0.79080900
C	1.69702100	-2.20631100	-2.14375000
H	0.17395200	-0.71284200	-1.97622200
C	2.95283700	-2.63638100	-1.71846900
H	4.62596800	-2.18139800	-0.44131600

H	1.14280300	-2.77649000	-2.88106000
C	-3.03394100	1.39999400	-0.62503600
C	-1.55851100	1.63999900	-0.65211000
C	-0.66127400	0.79356300	-0.07608300
H	3.38659100	-3.54826700	-2.11093700
C	3.99785300	0.03887900	0.69010100
O	3.71861700	1.35492900	0.75411200
O	4.89423200	-0.47807700	1.31671200
C	4.50049500	2.13050400	1.68143200
H	5.55331900	2.10855000	1.39734800
H	4.10528000	3.14165900	1.61679700
H	4.39108600	1.73588800	2.69207300

Compound 4

0 1

C	5.34265700	-1.50364600	-0.50890400
C	4.94874300	-0.24907100	-0.05510500
C	3.59532600	0.09863400	-0.06748300
C	2.63235400	-0.80959300	-0.53600800
C	3.03713800	-2.06386300	-0.99004800
C	4.38710900	-2.40890800	-0.97443800
H	6.39139000	-1.77682800	-0.50144400
H	5.67117500	0.47043200	0.31022200
H	2.28424900	-2.75178100	-1.35393800
H	4.69586500	-3.38555200	-1.32937300
C	1.17236000	-0.45155800	-0.58009100
O	0.37074000	-1.21244100	-1.08759100
O	3.92575200	2.27641500	0.85264200

O	1.41483500	2.95131700	0.89051200
H	2.26935000	3.35482300	1.13999900
S	-0.94097800	1.35671100	-0.06171900
C	-1.78501700	-0.02505400	0.70566200
C	-3.05470900	-0.41520700	0.26456100
C	-1.23678800	-0.68392700	1.81300000
C	-3.74533000	-1.44973200	0.89240900
C	-1.91414900	-1.72595600	2.43309000
H	-0.27107400	-0.36790800	2.18872600
C	-3.17135700	-2.11278900	1.97201500
H	-4.72433800	-1.73355400	0.52847100
H	-1.46525300	-2.22601100	3.28344300
C	3.16407400	1.41847600	0.42071500
C	1.70420300	1.73442300	0.42483500
C	0.75580000	0.85161800	0.00645500
H	-3.70952300	-2.92164900	2.45193100
Br	-3.90775100	0.46529200	-1.20854400

Compound 5

0 1

C	-5.15579500	2.39878000	0.25214000
C	-5.09102100	1.00916800	0.26619600
C	-3.87442700	0.37005300	0.01413800
C	-2.71878900	1.12130100	-0.25405900
C	-2.79426300	2.51308000	-0.26869300
C	-4.00840600	3.14811300	-0.01429100
H	-6.09755600	2.89843500	0.44697100
H	-5.96766600	0.40620500	0.46860600

H	-1.89668400	3.07950300	-0.48310900
H	-4.06102600	4.23079500	-0.02673200
C	-1.40336100	0.45575200	-0.55128700
O	-0.44442600	1.12068500	-0.89616800
O	-4.73967000	-1.84161100	0.25839600
O	-2.48523800	-3.07310900	-0.15018600
H	-3.41061800	-3.31898400	0.04578200
S	0.19245200	-1.87705400	-0.75691600
C	1.38937000	-0.96418300	0.22548700
C	2.56326900	-0.53736900	-0.39764800
C	1.20559300	-0.77165300	1.59630100
C	3.54354000	0.08085000	0.37134800
C	2.19112500	-0.12717400	2.33786300
H	0.30391600	-1.12863700	2.07863500
C	3.37282900	0.30111200	1.73414700
H	2.04864800	0.03037200	3.40099500
H	4.14468900	0.79178300	2.31226800
C	-3.78966900	-1.09938400	0.03568800
C	-2.46116400	-1.73881000	-0.19704300
C	-1.32469900	-1.02327600	-0.41919600
H	2.69710800	-0.66890800	-1.46275100
Br	5.16142400	0.66418300	-0.47865400

Compound 6

0 1

C	-4.81390000	-2.77803400	-0.29971400
C	-4.84486700	-1.43215200	-0.65080200
C	-3.78525700	-0.59716000	-0.28746600

C	-2.69196400	-1.10750700	0.43060700
C	-2.67146700	-2.45649000	0.78080700
C	-3.72797000	-3.28808200	0.41402500
H	-5.63355000	-3.42924100	-0.58011200
H	-5.67692300	-1.01273900	-1.20290200
H	-1.82533100	-2.83533300	1.34023500
H	-3.70609000	-4.33664000	0.68820100
C	-1.55206600	-0.22286300	0.85421400
O	-0.68226500	-0.65446300	1.58793700
O	-4.70718000	1.36646100	-1.28466100
O	-2.72466200	2.94220300	-0.68952800
H	-3.58104500	3.01309100	-1.15506700
S	-0.21753700	2.27873100	0.80489400
C	1.25085100	1.31093300	0.43448600
C	2.21079300	1.13088100	1.43040800
C	1.48803800	0.82748800	-0.85392400
C	3.40227600	0.46775100	1.14578000
C	2.66578700	0.14273100	-1.14124100
H	0.75781500	0.98795800	-1.63815800
C	3.61442100	-0.02732900	-0.13677400
H	4.14580000	0.32519700	1.91920400
H	2.84757000	-0.23825700	-2.13794600
C	-3.79890900	0.82628300	-0.66291700
C	-2.62336800	1.66942000	-0.29720000
C	-1.53628800	1.17928200	0.35941500
Br	5.24362600	-0.95796200	-0.53069200
H	2.02164200	1.49040600	2.43426600

Compound 7

0 1

C	4.51933000	-1.85775900	0.16861400
C	4.21827400	-0.51001200	0.33808700
C	2.91855500	-0.05562200	0.10154300
C	1.91604800	-0.95004700	-0.30626600
C	2.22776200	-2.29809000	-0.47566800
C	3.52458200	-2.74920700	-0.23705200
H	5.52658200	-2.21409300	0.35037500
H	4.97320600	0.20105500	0.65031700
H	1.44615100	-2.97506300	-0.79694200
H	3.76127200	-3.79861500	-0.37075500
C	0.51502000	-0.48137100	-0.58881900
O	-0.30780400	-1.25272200	-1.04524400
O	3.38858900	2.22290800	0.63752500
O	0.97152900	3.10683700	0.25090200
H	1.83456500	3.47777200	0.52007400
S	-1.43696300	1.57730400	-0.62799900
C	-2.48948200	0.35275000	0.14965200
C	-3.53330100	-0.22534800	-0.57011000
C	-2.35338900	0.01056500	1.49860500
C	-4.41405200	-1.12809600	0.00601900
C	-3.21775100	-0.90489300	2.09065300
H	-1.56309000	0.47025100	2.08021300
C	-4.25024100	-1.47293700	1.34457500
H	-5.20431800	-1.55137200	-0.60162100
H	-3.09294800	-1.16345600	3.13548700

H	-4.93149600	-2.17993800	1.80306000
F	-3.70467400	0.09496700	-1.86948600
C	2.58465300	1.36675000	0.28457000
C	1.17687000	1.80072500	0.05689400
C	0.18462900	0.94020800	-0.30168100

Compound 8

0 1

C	-4.49067300	-2.05812100	-0.29107800
C	-4.27247900	-0.68821600	-0.39709100
C	-3.01134900	-0.16307600	-0.10381600
C	-1.96522100	-1.00888500	0.29816000
C	-2.19430700	-2.37969100	0.40454500
C	-3.45211400	-2.90136100	0.10805900
H	-5.46747500	-2.46935900	-0.51753100
H	-5.06274800	-0.01371500	-0.70297400
H	-1.38023800	-3.01902300	0.72234700
H	-3.62435300	-3.96834600	0.19168100
C	-0.60634800	-0.46460300	0.64258400
O	0.24682800	-1.19728800	1.10720900
O	-3.60921000	2.10175000	-0.56514000
O	-1.26244400	3.11635700	-0.08422300
H	-2.14036600	3.44292800	-0.36307100
S	1.22282200	1.69503400	0.78885600
C	2.37281400	0.57647200	-0.02063500
C	3.45441400	0.09836000	0.71947800
C	2.25378200	0.27005600	-1.37878600
C	4.39689400	-0.68474300	0.07227700
C	3.20875700	-0.53912500	-1.98865200
H	1.42610300	0.66623500	-1.95375800
C	4.29909000	-1.02402200	-1.26755200
H	3.11313000	-0.78297200	-3.04043200
H	5.05820100	-1.64619200	-1.72412700

C	-2.76300900	1.28304400	-0.22320400
C	-1.38951200	1.79441100	0.05746900
C	-0.35449100	0.98214100	0.40680000
H	3.55433400	0.30512200	1.77689600
F	5.44882000	-1.15084600	0.78835300

Compound 9

0 1

C	-4.36559500	-2.20385800	-0.39704700
C	-4.18304300	-0.83880600	-0.59491300
C	-2.97599400	-0.24083000	-0.22437500
C	-1.94836300	-1.00821700	0.34670900
C	-2.14184400	-2.37432000	0.54444100
C	-3.34563300	-2.96915100	0.17113600
H	-5.30031100	-2.67170600	-0.68335900
H	-4.96015000	-0.22381600	-1.03186400
H	-1.34335200	-2.95237500	0.99240500
H	-3.49020500	-4.03224400	0.32631900
C	-0.65029000	-0.38163800	0.77614900
O	0.17182000	-1.03800000	1.38872000
O	-3.59990700	1.95457700	-0.92441100
O	-1.34549400	3.09827700	-0.30948700
H	-2.20241500	3.36526600	-0.69610400
S	1.09731200	1.85053200	0.89127000
C	2.34780400	0.71159700	0.27811800
C	3.25260400	0.14030900	1.17438600
C	2.47898300	0.47383800	-1.09238600
C	4.28535900	-0.66877500	0.70778600

C	3.49434500	-0.35210600	-1.56760600
H	1.79224200	0.93724700	-1.79032800
C	4.37992400	-0.90407100	-0.65473800
H	4.99681400	-1.12501800	1.38461100
H	3.61219600	-0.55519800	-2.62471200
C	-2.76463100	1.20069300	-0.43709000
C	-1.44291900	1.78745200	-0.07007800
C	-0.42010400	1.04789300	0.44007400
H	3.13839100	0.31263400	2.23690700
F	5.37609100	-1.70024300	-1.11290800

B3LYP / 6-311G(d,p) for C S H O; LanL2DZ for Ti

Neutral Compound 1

0 1

C	2.71858800	-1.34882300	0.00032300
C	1.37441700	-1.70355600	-0.00002000
C	0.39422900	-0.70748700	-0.00016700
C	0.76077600	0.64964100	-0.00005300
C	2.11010600	0.99378700	0.00016600
C	3.08448300	-0.00144800	0.00042200
H	3.48134800	-2.11864700	0.00050200
H	1.06429100	-2.74131500	-0.00019900
H	2.37233500	2.04449700	0.00012500
H	4.13326500	0.27291500	0.00070000
C	-0.28094600	1.73572800	-0.00025000
O	0.04726200	2.91197300	-0.00060600
O	-1.45199700	-2.22465400	-0.00098600

O	-3.31156700	-0.39235300	0.00100300
H	-3.26390200	-1.36789800	0.00003700
C	-1.03233900	-1.07538400	-0.00033600
C	-2.04577800	0.03070500	0.00039000
C	-1.68808200	1.33182400	0.00007700
H	-2.42963700	2.12080400	0.00023800

Neutral Compound 2

0 1

C	4.31456300	-1.82549700	0.40752100
C	3.98944500	-0.47859300	0.52441500
C	2.70975500	-0.04260600	0.17365400
C	1.75180000	-0.95365600	-0.29718100
C	2.08788600	-2.30069400	-0.41330700
C	3.36387300	-2.73408100	-0.06039400
H	5.30623100	-2.16835000	0.67868600
H	4.70810600	0.24792700	0.88304200
H	1.33923700	-2.98887600	-0.78498600
H	3.61923100	-3.78357400	-0.15242300
C	0.37425400	-0.50530600	-0.70533300
O	-0.39604100	-1.29429500	-1.21828700
O	3.11275200	2.24992800	0.70098800
O	0.73532500	3.10061000	0.10360500
H	1.57817500	3.47011600	0.43314000
S	-1.59900400	1.51373800	-0.92300400
C	-2.69948200	0.32932800	-0.13011900
C	-3.60368600	-0.38740400	-0.91398100

C	-2.71892500	0.19913600	1.26012800
C	-4.52802700	-1.23227200	-0.30461200
C	-3.63029500	-0.66535800	1.86061300
H	-2.02958200	0.77556100	1.86550400
C	-4.53955700	-1.37819300	1.08087900
H	-5.22945900	-1.78845200	-0.91626300
H	-3.63902400	-0.76991900	2.93985000
H	-5.25426700	-2.04360000	1.55162400
C	2.34982300	1.37858100	0.30004200
C	0.95923100	1.79408400	-0.05026500
C	0.00889400	0.91499900	-0.47013300
H	-3.56818900	-0.29539500	-1.99212300

Anionic Compound 1

-1 1

C	2.72559200	-1.26974200	0.00024800
C	1.39197600	-1.66590900	-0.00002400
C	0.37039400	-0.71182200	-0.00023600
C	0.68507200	0.65466400	-0.00018500
C	2.02551500	1.04434700	0.00006500
C	3.04049000	0.09141800	0.00029000
H	3.51655400	-2.01377800	0.00041700
H	1.10920900	-2.71265600	-0.00009600
H	2.22986600	2.10877700	0.00007000
H	4.08011100	0.40679900	0.00049200
C	-0.39483600	1.71961600	-0.00045100
O	-0.02930500	2.91263200	0.00016700

O	-1.33819000	-2.34518600	-0.00017400
O	-3.34401400	-0.47028200	0.00048700
C	-1.06108700	-1.15960800	-0.00057800
C	-2.16928000	-0.07647400	0.00008300
C	-1.73652200	1.27286800	-0.00004700
H	-2.50754500	2.03739900	0.00028000

$(TiO_2)_9$ Cluster

0 1

Ti	-2.11722700	-1.13106800	1.18955600
O	-1.71470100	-2.96530200	1.12979500
O	-1.66991000	-0.31255600	2.60981900
Ti	3.05112400	-2.39860300	-0.13389400
O	1.68690000	-3.47719400	0.38691800
O	1.08971400	0.64085200	3.40792500
O	2.62461600	-1.67477500	-1.64246700
Ti	-3.25127500	0.57938300	-0.98180300
O	-2.59399400	-0.18421800	-2.53735800
O	-2.75577700	2.25582000	-0.68694700
O	-3.72713700	-0.51410700	0.37550700
Ti	2.05621000	0.12957500	-0.88273900
Ti	1.92881800	2.92287900	-0.27102000
Ti	-0.07506900	-2.83964200	0.33978000
Ti	0.11476800	0.55523600	2.13189400
O	-0.60065100	-2.39507300	-1.39046400
O	-0.91237900	2.19893000	1.70017900
O	2.96486800	1.53485800	-1.26648300

O	2.73683000	3.79889800	0.82068100
O	2.88189600	-0.86657500	0.64911100
O	1.00200400	1.24343800	0.34521500
O	0.08927700	-1.21422300	0.98378800
Ti	-0.90984300	-0.73517300	-1.99805000
Ti	-0.94202300	2.24071800	-0.01904500
O	0.59120700	0.15840700	-2.14844600
O	0.16278600	3.48664700	-0.66565000
O	-1.45813000	0.14708400	-0.35148600

Neutral I lawsone-$(TiO_2)_9$

0 1

Ti	-2.18393300	-2.61506300	0.03961000
O	-1.49819100	-3.29011400	1.66276800
O	-3.80150400	-2.08386300	0.10539800
Ti	-0.33786100	1.07240000	3.66168500
O	-0.52344000	-0.72176900	3.84159500
O	-4.89951700	0.50491800	1.18773200
O	0.76671600	1.34235900	2.36401500
Ti	-0.78936800	-2.19178400	-2.49702500
O	0.95734800	-1.86739000	-2.03287100
O	-1.60184800	-0.91160500	-3.43245200
O	-1.70303600	-3.46341300	-1.57653200
Ti	-0.67001300	1.77711500	0.94964400
Ti	-2.15066700	3.15987600	-1.05439500
Ti	-0.78376900	-1.74143700	2.28285600
Ti	-3.66131300	-0.07975600	0.34010200

O	0.66963200	-1.62403500	1.11598000
O	-3.78288100	0.07794900	-1.63482700
O	-0.72272400	3.37740000	0.32889600
O	-3.42182300	4.16058300	-0.99642900
O	-1.63895400	1.68269800	2.69976400
O	-2.23334500	1.38021000	-0.12163700
O	-1.94701600	-0.80845500	1.36614300
Ti	0.86857700	-0.84586000	-0.47746700
Ti	-2.16788000	0.39319200	-2.14414900
O	0.47596000	0.90205300	-0.35413200
O	-1.94993900	2.11305500	-2.61101800
O	-1.16545800	-1.10562900	-0.96845400
C	3.88253400	1.61878700	-0.80769700
C	5.04365600	2.31540800	-0.90339700
C	6.35622300	1.72542400	-0.64455300
C	6.39822000	0.27667100	-0.26186000
C	5.20606600	-0.46712700	-0.17332200
C	3.92569500	0.17189600	-0.46582300
C	5.24809100	-1.82193900	0.18423000
C	6.47040900	-2.42614800	0.45454900
C	7.64998400	-1.68602300	0.36533900
C	7.61392300	-0.33752500	0.00715300
O	2.72065500	2.20495400	-1.06450100
H	5.01996700	3.36545500	-1.17028300
O	7.37957100	2.38433400	-0.72550400
O	2.87270800	-0.49656400	-0.43545900
H	4.32469600	-2.38412600	0.24710000

H	6.50434600	-3.47346900	0.73280700
H	8.60259000	-2.16072200	0.57465700
H	8.52007700	0.25177400	-0.06872700
H	1.91315800	1.69207900	-0.80999400

Neutral I 3-Thiomethyl-lawsone-$(TiO_2)_9$

0 1

Ti	3.84257700	1.72228700	-1.02751200
O	3.76269800	3.08416400	0.27530800
O	5.17532600	0.66871200	-0.88051500
Ti	1.67699700	0.37652500	3.83103300
O	2.47112200	1.92306200	3.31827400
O	5.56141100	-1.66007100	0.98357200
O	0.30424600	0.14944500	2.81100900
Ti	1.90454400	1.08281700	-3.11243100
O	0.28594200	1.61962100	-2.44187500
O	2.03070000	-0.63738300	-3.56879200
O	3.35762700	2.13150400	-2.80372400
Ti	1.19883300	-1.21857300	1.55246200
Ti	1.68676200	-3.62143900	0.10228700
Ti	2.71843900	2.20918200	1.47400000
Ti	4.43513500	-0.94717500	0.07847700
O	1.10264900	2.29698200	0.54519800
O	4.08693400	-1.75242700	-1.70295900
O	0.58845100	-2.82512200	1.56953000
O	2.52149900	-4.97220900	0.42350900
O	2.48080200	-0.95287000	3.07284400

O	2.55343000	-1.82199200	0.30496800
O	3.29300900	0.66164400	0.89220600
Ti	0.31998700	1.19607400	-0.62328500
Ti	2.38854000	-1.56963800	-1.93299500
O	0.16775200	-0.44267900	0.10535000
O	1.51621700	-3.12482300	-1.70890400
O	2.20863700	0.48041900	-1.32869400
C	-3.30581200	0.07404200	0.11208500
C	-4.65796400	-0.17645600	0.24187800
C	-5.61683900	0.94883500	0.45713300
C	-5.10372600	2.33752100	0.23956600
C	-3.73925900	2.56484200	-0.00080500
C	-2.81948700	1.42828400	-0.07208500
C	-3.26809300	3.86884300	-0.18114400
C	-4.15729400	4.93921700	-0.12219500
C	-5.51082100	4.71162800	0.11763800
C	-5.98383200	3.41070300	0.30099200
O	-2.47183800	-0.96437000	0.07264300
O	-6.75656700	0.76221300	0.82935400
O	-1.60273000	1.63988900	-0.32545800
H	-2.21355600	4.03245800	-0.36424200
H	-3.79143800	5.95022400	-0.26291800
H	-6.20153500	5.54650100	0.16470200
H	-7.03158400	3.21461600	0.49478900
H	-1.50684000	-0.74112200	0.07786700
S	-5.12378600	-1.85907300	0.35307100
C	-6.85116000	-1.91518100	-0.14564400

C	-7.20234100	-1.67668100	-1.47585800
C	-7.80725900	-2.34467500	0.77430000
C	-8.52657300	-1.83641600	-1.87397300
H	-6.44654600	-1.37825600	-2.19396200
C	-9.12595100	-2.51708900	0.36277600
H	-7.52482200	-2.52618900	1.80481700
C	-9.48847600	-2.25753100	-0.95737200
H	-8.80280500	-1.64604600	-2.90581900
H	-9.87174300	-2.84737500	1.07807200
H	-10.51770900	-2.38993700	-1.27379600

Neutral II lawsone-$(TiO_2)_9$

0 1

Ti	1.79502300	0.57715300	-2.25767200
O	0.94463700	2.28674800	-2.71788000
O	3.51462000	0.54989100	-2.14801500
Ti	1.22036400	3.67899500	1.66481200
O	0.81922000	4.11448300	-0.08468800
O	5.84221500	0.12071000	-0.27595200
O	0.10548100	2.43166400	1.98965300
Ti	0.29974400	-1.87915700	-2.16130500
O	-1.23217300	-1.34160100	-1.27039900
O	1.16011900	-3.42340900	-1.76442700
O	0.85967300	-0.63805100	-3.35908300
Ti	1.48988700	0.84958400	1.60847600
Ti	2.86349100	-1.49964900	2.42203300
Ti	0.32410700	2.66775600	-1.11487400

Ti	4.30425600	-0.29634500	-0.54975200
O	4.03348600	-2.17436800	-0.86599000
O	1.64183600	-0.10065700	3.04741100
O	4.14425900	-1.85371300	3.35257000
O	2.54400600	2.57744900	1.68484900
O	3.09890500	-0.15308800	1.02014100
O	1.33239500	1.36825400	-0.49737900
Ti	-1.59283400	-0.15714900	0.05050200
Ti	2.40334100	-2.73180000	-0.49788700
O	-0.17873700	-0.04047000	1.01270200
O	2.22843600	-2.89540100	1.25102800
O	1.51993400	-1.09016700	-1.03294800
C	-4.12895600	-0.95313700	1.14162200
C	-5.02501000	-1.61140300	1.91789600
C	-6.46564600	-1.50181100	1.67790800
C	-6.92888100	-0.63924200	0.53091700
C	-6.00442900	0.04219400	-0.28457200
C	-4.58524200	-0.10031000	0.01133600
C	-6.44779500	0.83167700	-1.35196000
C	-7.80975400	0.94533400	-1.60371400
C	-8.72636100	0.27324700	-0.79318100
C	-8.28818500	-0.51682800	0.26949700
O	-2.82244500	-0.96318900	1.28013300
H	-4.70218300	-2.23396700	2.74257100
O	-7.27846400	-2.08385200	2.37687100
O	-3.67793800	0.46120400	-0.63256500
H	-5.72028800	1.34120700	-1.97116600

H	-8.15884400	1.55359100	-2.42946400
H	-9.78803800	0.36367700	-0.99187300
H	-8.98532700	-1.04709900	0.90621800
O	-1.39161100	1.70769600	-0.82479100
H	-2.20894800	1.91665600	-1.29603400

Neutral II 3-Thiophenyl-lawsone-$(TiO_2)_9$

0 1

Ti	-3.11664800	-1.28390700	-1.67573500
O	-2.91236700	-3.18620100	-1.22875900
O	-4.69857600	-0.60584400	-1.57491200
Ti	-2.78559200	-2.17729100	3.26295800
O	-2.85266800	-3.48949400	1.96448600
O	-6.40024200	1.43402400	-0.14394700
O	-1.30862300	-1.39991800	2.91663000
Ti	-0.96906900	0.23241400	-2.84429100
O	0.47101400	-0.31374100	-1.82362000
O	-1.24154500	1.97571800	-3.26326500
O	-2.08299400	-1.14082200	-3.24965500
Ti	-2.19369100	0.18828200	1.80293400
Ti	-2.62674500	2.95749800	1.34600300
Ti	-2.14793300	-2.96515600	0.34234900
Ti	-4.88640000	1.10433600	-0.60817700
O	-0.20495900	-2.64763900	0.09558100
O	-4.13302400	2.38944500	-1.82487300
O	-1.79428800	1.68417100	2.57861400
O	-3.54502700	4.13237300	1.98620300

O	-3.69297500	-0.81243700	2.73737500
O	-3.51118200	1.29611600	0.80677100
O	-2.59600600	-1.26928700	0.23692600
Ti	0.70319800	-0.79276900	-0.08999000
Ti	-2.37189900	2.43718900	-1.80316300
O	-0.48268800	0.04960000	0.82611900
O	-1.83156700	3.32212500	-0.37427500
O	-2.13626500	0.54363300	-1.45716300
C	3.51341400	-0.50563800	0.34718700
C	4.69846200	0.13588100	0.61066000
C	5.97637600	-0.62356900	0.48035200
C	5.92446300	-1.97989200	-0.17434400
C	4.69937500	-2.59151100	-0.49292900
C	3.47171500	-1.85842700	-0.20573800
C	4.67287800	-3.85791400	-1.08249800
C	5.86870800	-4.51566000	-1.35470400
C	7.08441400	-3.91156500	-1.03626000
C	7.11315300	-2.64748900	-0.44532000
O	2.31875700	0.01557500	0.54684700
O	7.03053700	-0.19444700	0.90231900
O	2.32444700	-2.30949000	-0.43778300
H	3.71866800	-4.30827700	-1.32483200
H	5.85352500	-5.49671200	-1.81428400
H	8.01464900	-4.42622200	-1.24689800
H	8.04830300	-2.16794400	-0.18501000
S	4.62244000	1.75076700	1.29524500
C	6.10387000	2.56590100	0.67832100

C	6.24311500	2.82469000	-0.68670000
C	7.04228700	3.05052000	1.58889500
C	7.34433500	3.54537900	-1.14043900
H	5.49177000	2.47472000	-1.38450200
C	8.13013300	3.78706100	1.12795100
H	6.92844300	2.83912500	2.64467300
C	8.28669300	4.02984400	-0.23506300
H	7.45585000	3.74245500	-2.20060700
H	8.86067600	4.16249600	1.83527000
H	9.13729200	4.59982200	-0.59063400
H	0.41847800	-3.31815000	-0.21382800

Anionic lawsone-$(TiO_2)_9$

-1 1

Ti	1.62792700	-0.59202400	-2.24404900
O	0.65703500	0.57024900	-3.41339200
O	3.36616900	-0.57051200	-2.30933900
Ti	1.19937600	3.96204000	-0.32344500
O	0.51427000	3.52229200	-1.96664000
O	5.86535800	-0.07761500	-0.71669700
O	0.25037600	3.07127900	0.80654500
Ti	0.25567300	-2.67183800	-0.78297200
O	-1.23611500	-1.79192600	-0.20155800
O	1.22887900	-3.81441800	0.27479600
O	0.68849700	-2.23633800	-2.48157500
Ti	1.50983300	1.46735500	0.98342100
Ti	3.22327000	-0.07664500	2.69635700

Ti	0.05731800	1.71500900	-2.15915800
Ti	4.30959700	-0.54095200	-0.59274000
O	-1.43146800	1.08701800	-1.29502300
O	4.09405900	-2.33681700	0.08234100
O	1.91187700	1.34990600	2.67906000
O	4.55903800	0.13204600	3.61807100
O	2.58173700	3.02076200	0.00486000
O	3.27585800	0.34858300	0.81451600
O	1.27039400	0.94081200	-1.08479100
Ti	-1.55359600	-0.01351300	0.18175400
Ti	2.52383100	-2.56304500	0.86368700
O	-0.09797000	0.43626200	1.06254500
O	2.56744200	-1.87979700	2.50260000
O	1.52128000	-1.42344500	-0.30547500
C	-4.22050100	0.22683800	1.37584400
C	-5.14435100	0.50009700	2.33839000
C	-6.57127400	0.32509200	2.11401600
C	-7.01776600	-0.17704700	0.76232500
C	-6.08562300	-0.46844300	-0.24660900
C	-4.65069100	-0.27880100	0.02943900
C	-6.51272600	-0.93058200	-1.49430700
C	-7.87101200	-1.10256700	-1.73668200
C	-8.80015800	-0.81420600	-0.73501700
C	-8.37562300	-0.35370000	0.50900200
O	-2.92991500	0.36118600	1.49846100
H	-4.83490300	0.86171700	3.31102100
O	-7.40576200	0.57180900	2.97698400

O	-3.76657600	-0.51165000	-0.78768500
H	-5.77008700	-1.14651900	-2.25271900
H	-8.20703100	-1.46046300	-2.70325700
H	-9.85911300	-0.94933300	-0.92640200
H	-9.07727400	-0.12350100	1.30129900

Anionic 3-thiophenyl-lawsone-$(TiO_2)_9$

-1 1

Ti	2.92859100	2.37505700	-0.97355500
O	2.28366800	3.52062200	0.39067100
O	4.60731000	2.01383500	-0.93947100
Ti	1.80396700	0.06599600	3.82674200
O	1.83733800	1.83634200	3.41462300
O	5.96147700	-0.02685200	0.76836500
O	0.54973300	-0.65561200	2.89278400
Ti	1.31136000	0.98278200	-2.85589500
O	-0.33794100	0.75254500	-2.04454100
O	2.08769900	-0.46396900	-3.60695100
O	2.10740500	2.62061900	-2.66600500
Ti	1.78300500	-1.40753800	1.40420200
Ti	3.18083200	-3.39690800	-0.10083100
Ti	1.76460000	2.23109600	1.56471100
Ti	4.60548700	0.17090600	-0.09196800
O	0.17548200	1.67990400	0.83983500
O	4.65739300	-0.69092000	-1.89597600
O	1.84488200	-3.13917400	1.33016400
O	4.42407800	-4.41183900	0.20337000

O	3.01090500	-0.76272500	2.92433500
O	3.29372200	-1.43100900	0.13545700
O	2.94117400	1.11392000	0.87961900
Ti	-0.57671100	0.43569200	-0.25668400
Ti	3.01126400	-1.20098700	-2.09018200
O	0.46622600	-0.95836900	0.16071400
O	2.81950200	-2.99708100	-1.90006000
O	2.24016400	0.54138300	-1.35519500
C	-3.46190100	0.31079700	0.23921100
C	-4.67016600	-0.26116700	0.55473200
C	-5.88862700	0.56562100	0.65423500
C	-5.79686100	2.01576400	0.24679100
C	-4.57522700	2.59734600	-0.12065000
C	-3.36863100	1.75796200	-0.12722200
C	-4.50719500	3.94662400	-0.47601900
C	-5.66392000	4.71844100	-0.46605100
C	-6.88246900	4.14298000	-0.10138500
C	-6.94962600	2.79772300	0.25409400
O	-2.31644900	-0.30346900	0.20741800
O	-6.95305400	0.12496600	1.05585200
O	-2.25832300	2.17396200	-0.44605300
H	-3.54741900	4.36556200	-0.75248400
H	-5.61745600	5.76616000	-0.74027300
H	-7.78335000	4.74665300	-0.09251100
H	-7.88310100	2.33272500	0.54530300
S	-4.72208500	-1.96975200	1.03391000
C	-6.07440300	-2.60172500	0.03065300

C	-6.03685800	-2.50553200	-1.36284900
C	-7.12563200	-3.27733100	0.65211400
C	-7.05861300	-3.06450900	-2.12523300
H	-5.20651200	-2.00251800	-1.84399800
C	-8.13227600	-3.85532500	-0.11774000
H	-7.15988300	-3.33072700	1.73324200
C	-8.10623200	-3.74509800	-1.50625100
H	-7.02595700	-2.98186400	-3.20619300
H	-8.94701800	-4.37823800	0.37144600
H	-8.89589400	-4.18784300	-2.10318700

3. Uv-vis spectra

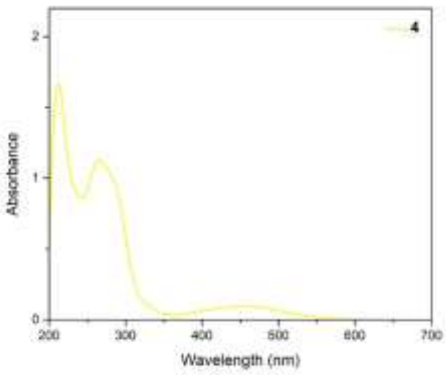

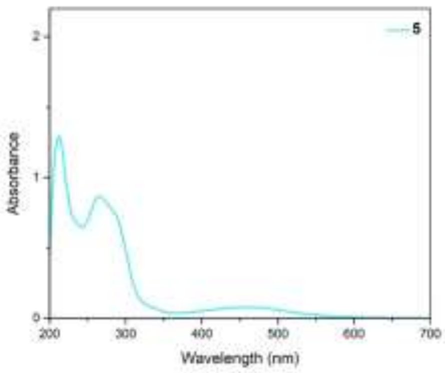

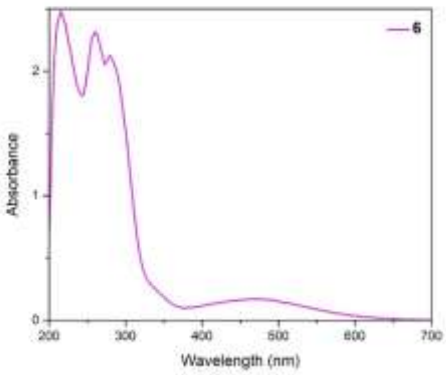

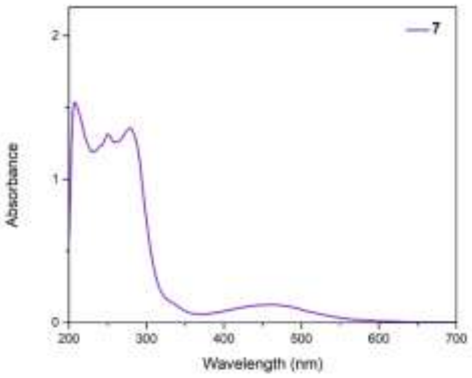

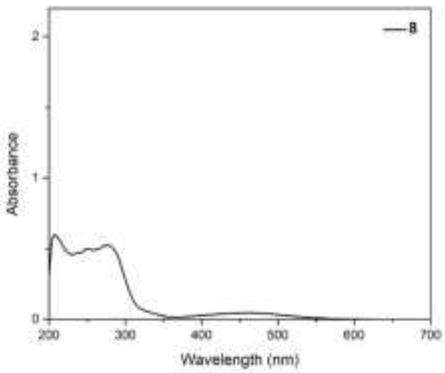

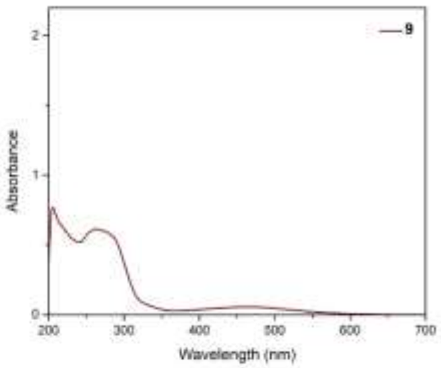

Supplement: Supplementary file 1 [file materials-14-05587-s001.zip › materials-1339069-supplementary.pdf]
